# Wave-swept coralliths of Saba Bank, Dutch Caribbean

**DOI:** 10.1007/s12526-017-0712-5

**Published:** 2017-05-03

**Authors:** Bert W. Hoeksema, Dahlia Hassell, Erik H. W. G. Meesters, Fleur C. van Duyl

**Affiliations:** 1 Naturalis Biodiversity Center, P.O. Box 9517, 2300 RA Leiden, The Netherlands; 2 Institute of Biology Leiden, Leiden University, PO Box 9505, 2300 RA Leiden, The Netherlands; 3 Saba Conservation Foundation, Fort Bay, Saba, Dutch Caribbean The Netherlands; 4 Wageningen University and Marine Research, P.O. Box 57, 1780 AB Den Helder, The Netherlands; 5 NIOZ Royal Netherlands Institute for Sea Research and Utrecht University, P.O. Box 59, 1790 AB Den Burg, Texel The Netherlands

**Keywords:** Reef corals, Ecophenotypic variation, Free living, Rotatory, Anthocyathus, Spheroidal, Tumbleweed, Discoidal

## Abstract

During a recent reef coral survey at the submarine Saba Bank (Eastern Caribbean), an uncommon and diverse assemblage of unattached scleractinian corals (coralliths) was encountered, which has not been reported from the Atlantic before. Four different types of these free-living (unattached) corals were distinguished. They were observed on a relatively flat seafloor (15–20 m deep) with poor coral cover and full exposure to oceanic swell. Much of the substratum was not consolidated and consisted mainly of sand and fragments of branching coralline algae. One of the four types is the (1) anthocyathus stage in the life history of the free-living species *Manicina areolata* and *Meandrina danae*. The other three are coralliths formed as ecophenotypic varieties: (2) spheroidal–amoeboidal (= globular and (sub)massive) in *Porites astreoides*, *Siderastrea radians*, *S. siderea*, and *Stephanocoenia intersepta*; (3) tumbleweed-like (= globular and ramose) in *Porites divaricata* and *P. furcata*; and (4) discoidal (flat and circular with short branches) in *Madracis decactis* and possibly in *M.* cf. *auretenra*. This assemblage of free-living corals is likely related to a combination of abiotic factors consisting of wave exposure (swell), depths that waves can reach, a horizontal sea floor with little relief, an unconsolidated substratum, and low coral cover.

## Introduction

**Fig. 1:**
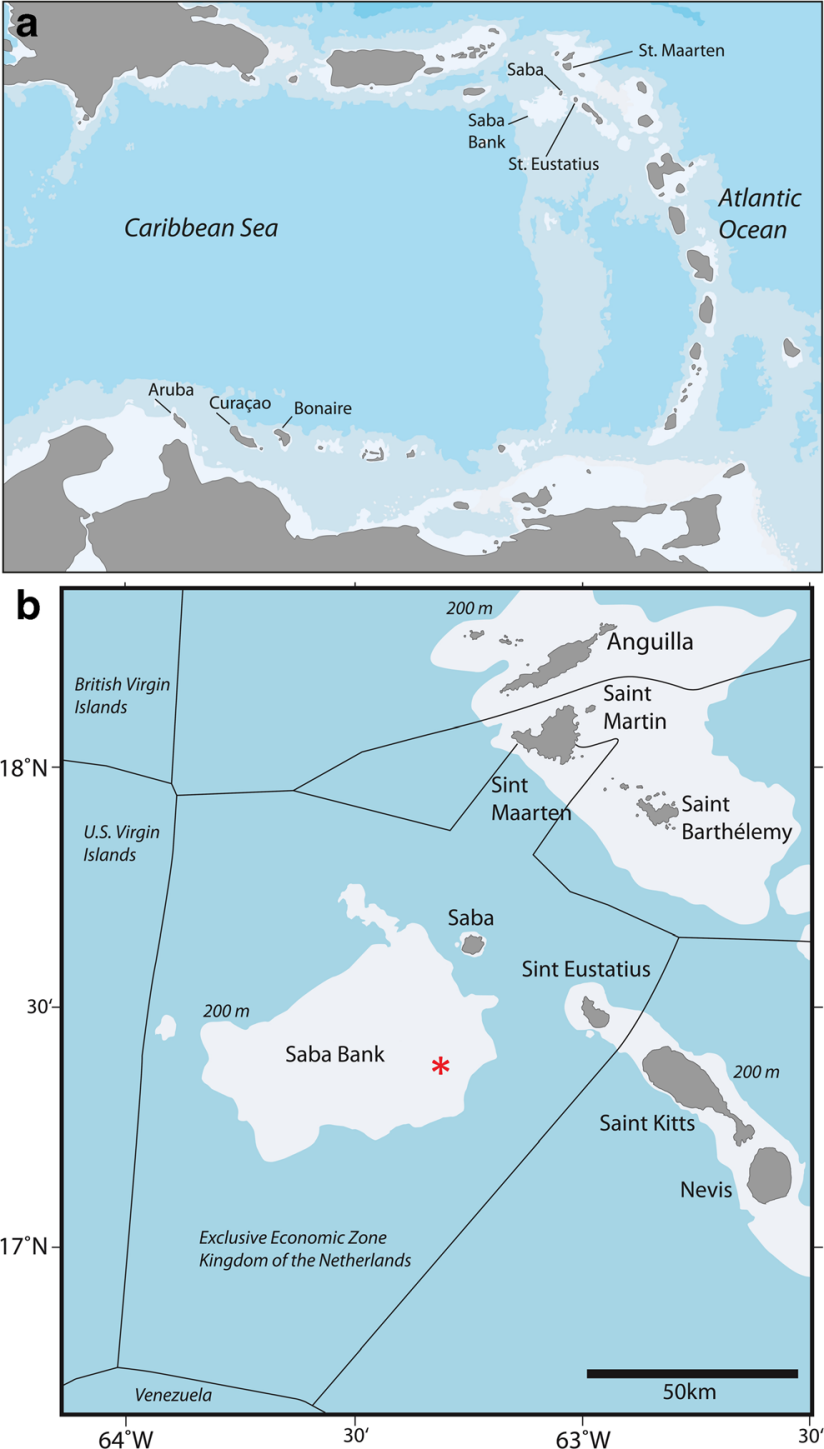
**a** The position of Saba Bank in proximity to the Dutch Caribbean islands Saba, St. Eustatius, and St. Maarten. **b** The position of research site Tertre de Fleur (*asterisk*, 17° 23′ 04″ N, 63° 17′ 23″ W)

Saba Bank is a large submarine carbonate platform located to the west of the volcanic islands Saba and St. Eustatius, eastern Caribbean, at roughly 17° 30′ N, 63° 30′ W (Fig. 1). It is 65 km long and 40 km wide. Its upper surface is generally flat and slightly tilting (practically horizontal) with depths in eastward direction generally rising from 50 to 15 m. The southeastern ridge reaches up to 12 m depth (Chart 2020, Royal Netherlands Navy), although one source mentions 7 m (Macintyre et al. 1975). This ridge is more exposed to currents than other parts of the bank, while the presence of sand waves on the western part of Saba Bank at 30–40 m depth suggests that the whole platform is wave-swept because of swell (Van der Land 1977). Owing to these conditions, the Saba Bank is not always accessible by divers using SCUBA (self-contained underwater breathing apparatus), which hampers scientific research.

The stony coral fauna (Scleractinia, Milleporidae, Stylasteridae) of Saba Bank was studied during two exploratory expeditions by use of SCUBA, in 1972 (Van der Land 1977) and in 2006 (McKenna and Etnoyer 2010). Coral specimens were sampled as reference material for identification, and they were deposited in museum collections for follow-up studies (Hoeksema et al. 2011). During an earlier expedition in 1971, corals were dredged from 15 to 100 m depths but this did not result in other species records (Macintyre et al. 1975). Stony coral species were also mentioned in a report of a post-hurricane assessment in 1999 (Klomp and Kooistra 2003) and in a report on a benthic survey that took place in 2007 (Toller 2008), but these did not include any remarkable records for Saba Bank. The stony corals of Saba Bank were also subject of more extensive surveys in 1996, 2011, and 2013, which eventually resulted in a total record of 39 species (Meesters et al. 1996; Van Beek and Meesters 2013, 2014).

In addition to bottom depth and substrate, wave energy may be a major factor influencing the variation in species composition on Saba Bank (Van der Land 1977; McKenna and Etnoyer 2010). Water movement can also affect the shape of corals (Todd 2008), but earlier Saba Bank studies did not take that into account and only remarked on the relatively small size of the corals (Macintyre et al. 1975; Van der Land 1977). Available coral collections from Saba Bank (Hoeksema et al. 2011) did not reveal clear ecophenotypic variation as observed in various coral collections from elsewhere (e.g., Wijsman-Best 1974; Best et al. 1984; Hoeksema and Moka 1989; Hoeksema 1993; Amaral 1994; Gittenberger and Hoeksema 2006; Sorauf and Harries 2010; Hoeksema 2012a).

During a recent reef coral survey (November 2015) on Saba Bank, some scleractinian corals were found on unconsolidated substratum and appeared to be free-living (unattached) instead of attached. Stony corals may become detached from their original substratum in various ways, and as such, they are less commonly known from reefs in the Atlantic than in the Indo-Pacific (Hoeksema 1993). Corals of some species developed free-living life history traits, which are most common in the Indo-Pacific, where over 45 of such species are recognized (Best and Hoeksema 1987; Hoeksema 1993; Gittenberger et al. 2011; Benzoni et al. 2012). In the Atlantic, only three recent free-living shallow-water coral species are known, i.e., *Manicina areolata* (Linneaus, 1758), *Meandrina danae* (Milne-Edwards and Haime, 1848), and *Meandrina brasiliensis* (Milne-Edwards and Haime, 1848), forming a remnant of a richer Neogene free-living scleractinian fauna (Klaus et al. 2011, 2013; Pinzón and Weil 2011; Meesters et al. 2013). Free-living corals may also become detached after budding, as seen in most mushroom corals (Kramarsky-Winter and Loya 1996; Gilmour 2004; Hoeksema 2004; Hoeksema and Yeemin 2011) and *Goniopora stokesi* Milne Edwards and Haime, 1851 on Indo-Pacific reefs (Boschma 1923; Hoeksema and Waheed 2011a), and as observed in various species of deep sea corals (Cairns 1988). Small corals belonging to the genera *Heteropsammia* (Dendrophylliidae) and *Heterocyathus* (Caryophylliidae) become free living by entirely overgrowing and incorporating gastropod shells inhabited by a sipunculan worm of the genus *Aspidosiphon* (Hoeksema and Best 1991; Hoeksema and Matthews 2015; Igawa et al. 2017). Both coral genera are traditionally known from the Indo-Pacific, but *Heterocyathus* has recently also been found in the Caribbean (Reyes et al. 2009; Santodomingo et al. 2013). ﻿Corals that are usually attached to the substrate may become detached by mechanical force or overgrow loose substrate and continue to live as so-called coralliths with a subspherical, globular, or amoeboidal growth form (Glynn 1974; Dullo and Hecht 1990; Roff 2008; Capel et al. 2012; Tortolero-Langarica et al. 2016), also known as circumrotatory or just rotatory corals (Kissling 1973; Sorauf and Harries 2009; Sorauf 2010). The shape of unattached reef corals can also be affected by fragmentation, a means of asexual reproduction that has been observed on Indo-Pacific reefs (Littler et al. 1997; Yamashiro and Nishihira 1998; Feingold 2001; Hoeksema and Gittenberger 2010; Hoeksema and Waheed 2011b), in South Atlantic rock pools (Hoeksema 2012a; Hoeksema and Wirtz 2013), and in deep waters (Cairns 1988). Unattached corals are known to be mobile, in a passive way either by external force (Glynn 1974; Jokiel and Cowdin 1976; Hubman et al. 2002) or by auto-locomotion (Chadwick-Furman and Loya 1992; Yamashiro and Nishihira 1995; Hoeksema and De Voogd 2012; Hoeksema and Bongaerts 2016). All examples above concern scleractinian species (Anthozoa), but fire corals (Hydrozoa: Milleporidae) have also been observed to become detached and continue life as free-living corals, both in the Caribbean (Edmunds 1999; Castro et al. 2006) and in the Indo-Pacific (Razak and Hoeksema 2003).

The free-living coral fauna observed on Saba Bank appeared to consist of corals that were detached either by life history strategy as anthocyathus stage, which is free living as opposed to the attached anthocaulus stage (Wells 1966; Hoeksema 1989: Fig. 42), or by corallith forming after detachment from the substrate. Some of them belonged to species previously unreported to become free living, and one of the observed corallith shapes has not been described before. The present report serves to document this unique assemblage of free-living corals, which may be related to Saba Bank’s physical setting as a wave-swept environment.

## Material and methods

The stony coral fauna (Scleractinia, Milleporidae, Stylasteridae) of Saba Bank was surveyed during three SCUBA dives using the roving diver or timed-swim method (ca. 1 h) at 15–25 m depths in November 2015. This is an ideal method for the in situ recording of as many coral species as possible over a large area in a limited time frame, including free-living and small species (Hill and Wilkinson 2004; Hoeksema and Koh 2009). All three dives were at a locality called Tertre de Fleur (17° 23′ 04″ N, 63° 17′ 23″ W), which is situated at the southeastern side of Saba Bank (Fig. 1b).

**Fig. 2:**
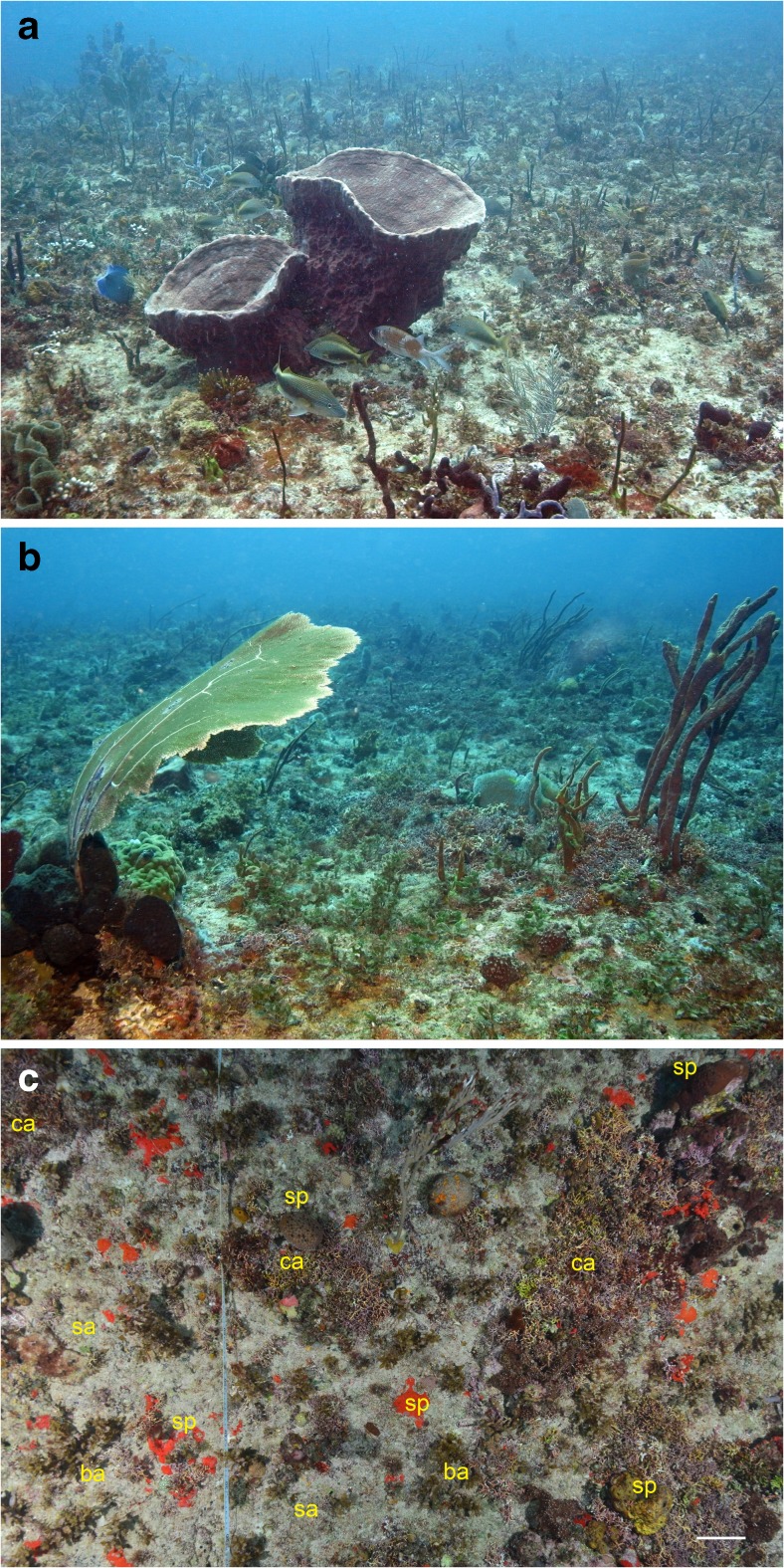
The sea floor at the research site Tertre de Fleur showing low coral cover and only a few large sedimentary animals, like sponges (**a**) and octocorals (**b**), among relatively small sessile invertebrates and algae; **c** 1.5 m^2^ surface area viewed from above with cover predominantly consisting of sand (*sa*), branching coralline algae (*ca*), sponges (*sp*), and brown algae (*ba*; *scale bar* 10 cm)

Tertre de Fleur is a disc-like hard bottom carbonate outcrop with a diameter of 600–700 m surrounded by sandy substratum with rubble (21 m depth). The outcrop is slightly elevated (up to 14 m depth) in the centre and smoothly drops to the surrounding sandy plains. It is located approximately 4 km from the steep, swell-exposed eastern edge of the bank (Fig. 1b). The outcrop itself is characterized by a low-relief hard-bottom rugosity colonized by fleshy brown and calcareous algae, sponges, and some corals (Fig. 2). It has a slightly undulating bottom which is interspersed with sand patches and shallow troughs with sand and rubble. Branching red calcareous algae are locally very abundant on Saba Bank (see Van Beek and Meesters 2013). Such algae are brittle, break easily, and become detached, forming maerl deposits and rhodoliths (Peña et al. 2014; Brasileiro et al. 2016; Riosmena-Rodríguez 2017; Sletten et al. 2017). Rhodoliths have been reported earlier from Saba Bank, but they were not taxonomically identified (Littler et al. 2010).

The sea floor at the dive site was predominantly flat with scarce coral growth. The substratum was not consolidated and consisted mainly of sand (Fig. 2). The stony coral cover (Scleractinia and Milleporidae) measured in 2011 and 2013 at 16 m depth was 2.6 and 1.2%, respectively, which was low compared to 10 other Saba Bank sites (17–30 m deep) with coral cover ranging 3.8–15.6% and 3.1–15.0% in the same years (Van Beek and Meesters 2014). Free-living corals were recorded with their depth, identified, and photographed. Because the number of dives at this site was limited to three during the 2015 survey, there was not sufficient time for the measurement of their densities.

Historical collection material at Naturalis Biodiversity Center (catalogued as RMNH Coel.) was used to verify earlier species records (Van der Land 1977). Coral identifications are based on information given by two field guides (Bright and Lang 2013; Humann and DeLoach 2013).The nomenclature of species mentioned in earlier records is updated according to the World Register of Marine Species (WoRMS; Hoeksema and Cairns 2015).

## Results and discussion

The total number of stony coral species (Scleractinia, Milleporidae, Stylasteridae) recorded from Saba Bank is nearly 50 (Table 1). Records of various observation years (Table 1) cannot directly be compared with each other because some concern species names that are currently unaccepted (Hoeksema and Cairns 2015) (like *Colpophyllia breviserialis* and *Dichocoenia stellaris*), Brazilian species that previously have not been recorded in the Caribbean (like *Favia leptophylla* and *M. brasiliensis*), and species preferring shallow depths (see Roos 1971; Bak 1975) that probably do not occur at Saba Bank, such as *Favia fragum*, which may resemble juvenile *Dichocoenia stokesii*. Such records could not be verified without access to collected specimens or photographic documentation. Some congeneric species (e.g., within either *Mycetophyllia* or *Scolymia*) may be difficult to distinguish from each other and even from species of related genera (e.g., Wells 1964; Fenner 1993; Budd et al. 2012), which may have resulted in records of misidentified specimens and overestimated species numbers. On the other hand, some small azooxanthellate species may have been overlooked and remained unrecorded, such as two caryophylliids recently found at nearby St. Eustatius (Fig. 1), *Colangia immersa* Pourtalès, 1871 and *Rhizosmilia maculata* (Pourtalès, 1874) (Hoeksema and Van Moorsel 2016).

**Table 1:** Stony coral species recorded from Saba Bank

Year of record	2015	1972	1996	2006	2011	2013
	A/U					
Scleractinia						
Acroporidae						
*Acropora cervicornis* (Lamarck, 1816)		a	d	e	f	
Agariciidae						
*Agaricia agaricites* (Linnaeus, 1758)	A	a	d	e	f	g
*Agaricia fragilis* Dana, 1848					f	g
*Agaricia grahamae* Wells, 1973				e	f	g
*Agaricia humilisa* (Verrill, 1901)	A			e	f	g
*Agaricia lamarcki* Milne Edwards and Haime, 1851			d	e	f	
*Agaricia tenuifolia* Dana, 1848	A					
*Helioseris cucullata* (Ellis and Solander, 1786)		b	d4	e	f	
Astrocoeniidae						
*Stephanocoenia intersepta* (Lamarck, 1816)	AU	a	d5	e	f	
*Madracis asperula* Milne Edwards and Haime, 1849		a				
*Madracis auretenra* Locke, Weil and Coates, 2007	U?		d6	e	f6	
*Madracis decactis* (Lyman, 1859)	A	c	d	e	f	g
Dendrophylliidae						
*Tubastraea coccinea* Lesson, 1829				e		
Meandrinidae						
*Dendrogyra cylindrus* Ehrenberg, 1834	A	a	d	e	f	
*Dichocoenia stellaris* Milne Edwards and Haime, 1848						g10
*Dichocoenia stokesii* Milne Edwards and Haime, 1848	A	a	d	e	f	g
*Eusmilia fastigiata* (Pallas, 1766)		a	d	e	f	g
*Meandrina brasiliensis* (Milne Edwards and Haime, 1848)				e11		
*Meandrina danae* (Milne Edwards and Haime, 1848)	U					
*Meandrina meandrites* (Linnaeus, 1758)	A	c	d	e	f	g
Merulinidae						
*Orbicella annularis* (Ellis and Solander, 1786)		a1	d1	e1	f1	g
*Orbicella faveolata* (Ellis and Solander, 1786)	A		d1	e7	f7	g
*Orbicella franksi* (Gregory, 1895)			d1	e8	f8	g
Montastraeidae						
*Montastraea cavernosa* (Linnaeus, 1767)	A	a	d	e	f	g
Mussidae						
*Colpophyllia breviserialis* M. Edwards and Haime, 1849						g10
*Colpophyllia natans* (Houttuyn, 1772)		a	d	e	f	g
*Diploria labyrinthiformis* (Linnaeus, 1758)	A	a	d	e	f	g
*Favia fragum* (Esper, 1795)				e	f	g
*Favia leptophylla* (Esper, 1795)						g11
*Isophyllia rigida* (Dana, 1846)		a	d	e9		g
*Isophyllia sinuosa* (Ellis and Solander, 1786)		a	d	e	f	g
*Manicina areolata* (Linnaeus, 1758)		a		e	f	
*Mycetophyllia aliciae* Wells, 1973					f	g
*Mycetophyllia danaana* Milne Edwards and Haime, 1849			d			g
*Mycetophyllia ferox* Wells, 1973						
*Mycetophyllia lamarckiana* Milne Edwards and Haime, 1848		a				g
*Mussa angulosa* (Pallas, 1766)		a		e	f	
*Mussismilia hispida* (Verrill, 1901)						
*Pseudodiploria clivosa* (Ellis and Solander, 1786)		a2			f2	
*Pseudodiploria strigosa* (Dana, 1846)	A	a3	d3	e3	f3	g3
*Scolymia cubensis* (Milne Edwards and Haime, 1848)					f	g
*Scolymia lacera* (Pallas, 1766)		a				
*Scolymia wellsii* Laborel, 1967						g
Oculinidae						
*Oculina varicosa* Le Sueur, 1820						g
Poritidae						
*Porites astreoides* Lamarck, 1816	AU	a	d	e	f	g
*Porites divaricata* Le Sueur, 1820	AU		d	e	f	g
*Porites furcata* Lamarck, 1816	AU				g	g
*Porites porites* (Pallas, 1766)	A	a	d		f	g
Siderastreidae						
*Siderastrea radians* (Pallas, 1766)	AU	a			f	g
*Siderastrea siderea* (Ellis and Solander, 1768)	AU		d	e	f	g
Scleractinia incertae sedis						
*Solenastrea bournoni* Milne Edwards and Haime, 1849		a				
*Solenastrea hyades* (Dana, 1846)						g
Hydrozoa						
Milleporidae						
*Millepora alcicornis* Linnaeus, 1758	A	a	d	e	f	g
*Millepora complanata* Lamarck, 1816			d		f	g
*Millepora squarrosa* Lamarck, 1816		a	d			g
Stylasteridae						
*Stylaster roseus* (Pallas, 1766)				e		

Records from 2015 survey: A, attached; U, unattached. Historical records: a, 1972 collection record (Van der Land 1977); b, 1972 overlooked collection record (Hoeksema et al. 2017); c, 1972 other collection material that was misidentified or overlooked; d, 1996 observation record (Meesters et al. 1996); e, 2006 collection record (McKenna and Etnoyer 2010); f, 2011 observation record (Van Beek and Meesters 2013); g, 2013 observation record (Van Beek and Meesters 2014). Notes: recorded as 1, *Montastraea* *annularis*; 2, *Diploria clivosa*; 3, *Diploria strigosa*; 4, *Leptoseris cucullata*; 5, *Stephanocoenia michelinii*; 6, *Madracis mirabilis*; 7, *Montastraea faveolata*; 8, *Montastraea franksi*; 9, *Isophyllastrea rigida*; 10, unaccepted name due to synonymy; 11, not Caribbean but Brazilian

In the present survey, 20 species of scleractinian corals were recorded on Saba Bank with a note mentioning whether they were observed attached or unattached (Table 1). The unattached form was either a coral represented by a free-living anthocyathus stage or a detached coral belonging to as species that is normally attached. Some species have not been recorded before in unattached form. Those represented by coralliths could be categorized in three different ecomorphs, one of which was not distinguished before.

**Fig. 3:**
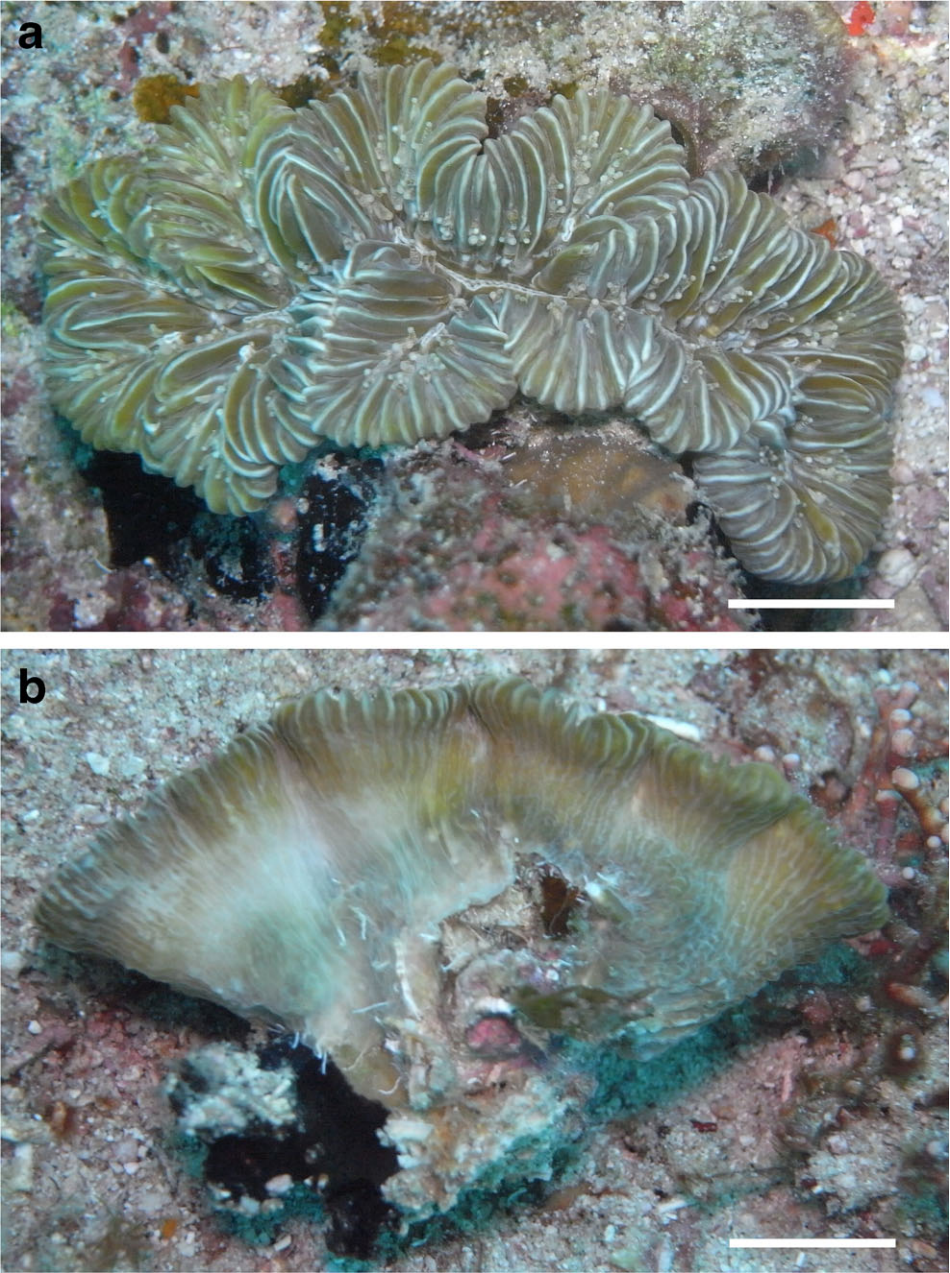
Anthocyathus stage of a *Meandrina danae* coral from above (**a**) and lateral view (**b**), showing detached condition. Locality: Tertre de Fleur, Saba Bank. *Scale bars* 1 cm

*M. danae* was represented by a single specimen in anthocyathus stage (Table 2, Fig. 3). It was previously recorded from Saba Bank only once, as *M. brasiliensis*, by McKenna and Etnoyer (2010), a species that is actually an endemic of Brazil (Pinzón and Weil 2011). *M. danae* has recently been recorded from the nearby island of St. Eustatius (Hoeksema and Van Moorsel 2016). This species may be rare, but because of its relatively small size, it can easily be overlooked or it can be confused with juveniles of its congener *M. meandrites*.

**Table 2:** Stony coral species of Saba Bank that show a free-living anthocyathus stage or a corallith shape

Corallith species	Anthocyathus	Spheroidal, massive	Spheroidal, ramose	Discoidal, branched	Illustration
*Meandrina danae*	x				Fig. 3
*Manicina areolata*	x				Fig. 4
*Porites astreoides*		x			Fig. 5a
*Siderastrea radians*		x			Fig. 5b
*Siderastrea siderea*		x			Fig. 5c, d
*Stephanocoenia intersepta*		x			Fig. 5f, g
*Porites divaricata*			x		Fig. 6a, c–e
*Porites furcata*			x		Fig. 6b, f, g
*Madracis decactis*				x	Fig. 7a–h
*Madracis* cf. *auretenra*				x	Fig. 7i, j

**Fig. 4:**
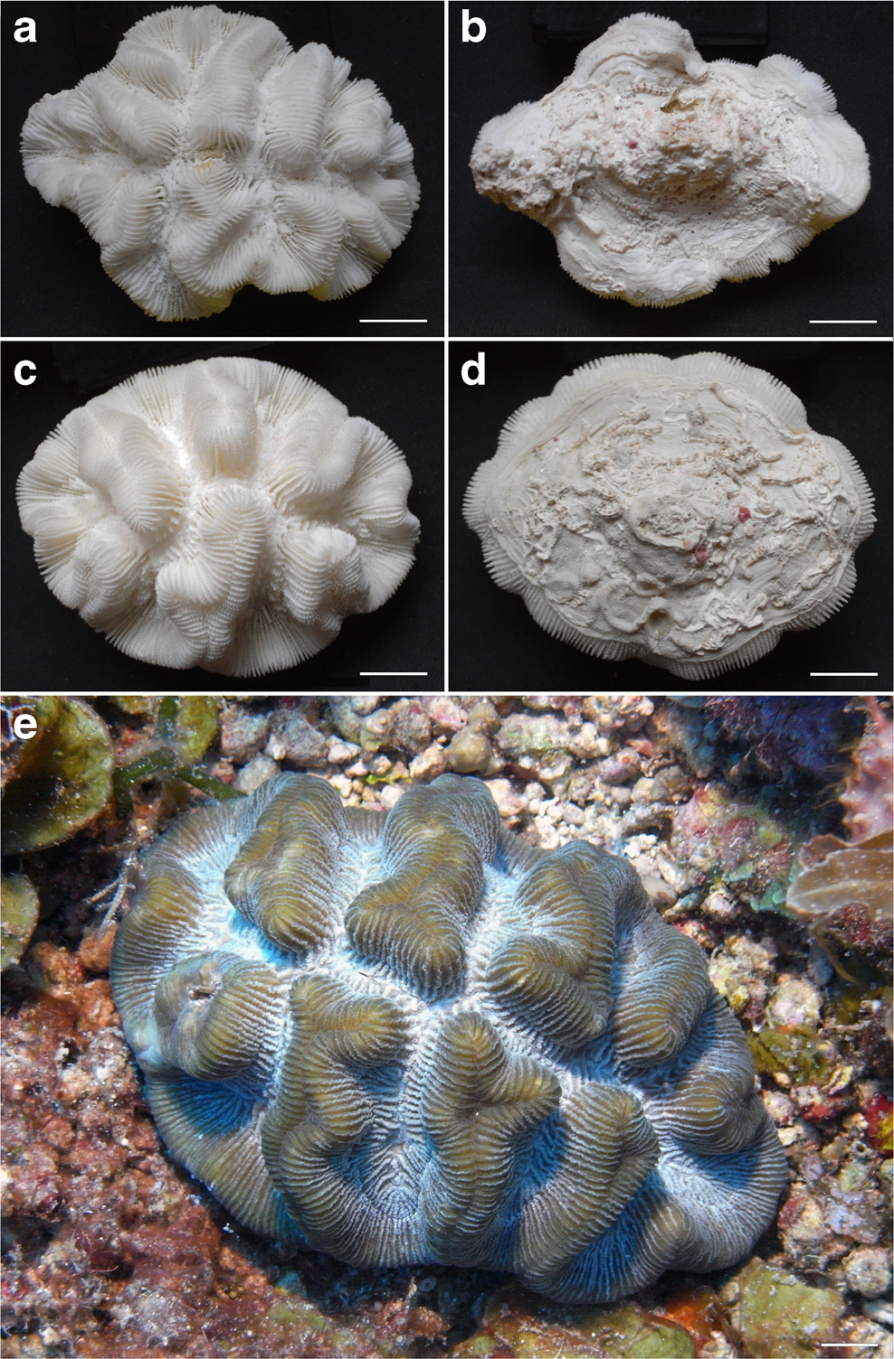
Anthocyathus stage of *Manicina areolata.* Two corals collected from Saba Bank in 1972 (Van der Land 1977), showing upper and lower surface. **a**, **b** RMNH Coel. 8701 from 22 m depth (Sta. 142, 17° 27′ N, 63° 21′ W). **c**, **d** RMNH Coel. 8650 from 21 m depth (Sta. 46, 17° 30′ N, 63° 28′ W). **e** Live specimen off St. Eustatius at 30 m depth on sea floor next to Charles L. Brown shipwreck (17° 27′ 51″ N, 62° 55′ 36″ W), 9 June 2015. *Scale bars* 1 cm

Another Caribbean coral with a free-living anthocyathus phase, *M. areolata*, was not found in 2015, but it was encountered during three previous surveys (Table 1, Fig. 4a–d). In the Saba Bank coral collection of 1972, 33 specimens were present that were collected from 21 to 41 m depths at 12 out of 17 sampling stations. One of these stations (Sta. 142 in Van der Land 1977) was relatively close to the present study site. These large numbers of corals and sampling sites in 1972 suggest that the species was common at that time or that it was a preferred collecting item. Whether the species has become less common at Saba Bank since 1972 is not certain. In 2015, a large specimen of the same species was encountered on coarse sediment at 30 m depth off the adjacent island of St. Eustatius (Fig. 4e). This species has been subject of various studies dealing with its morphology in relation to mobility and sediment rejection (Fabricius 1964; Johnson 1988; Hubman et al. 2002; Uhrin et al. 2005; Sorauf and Harries 2010).

**Fig. 5:**
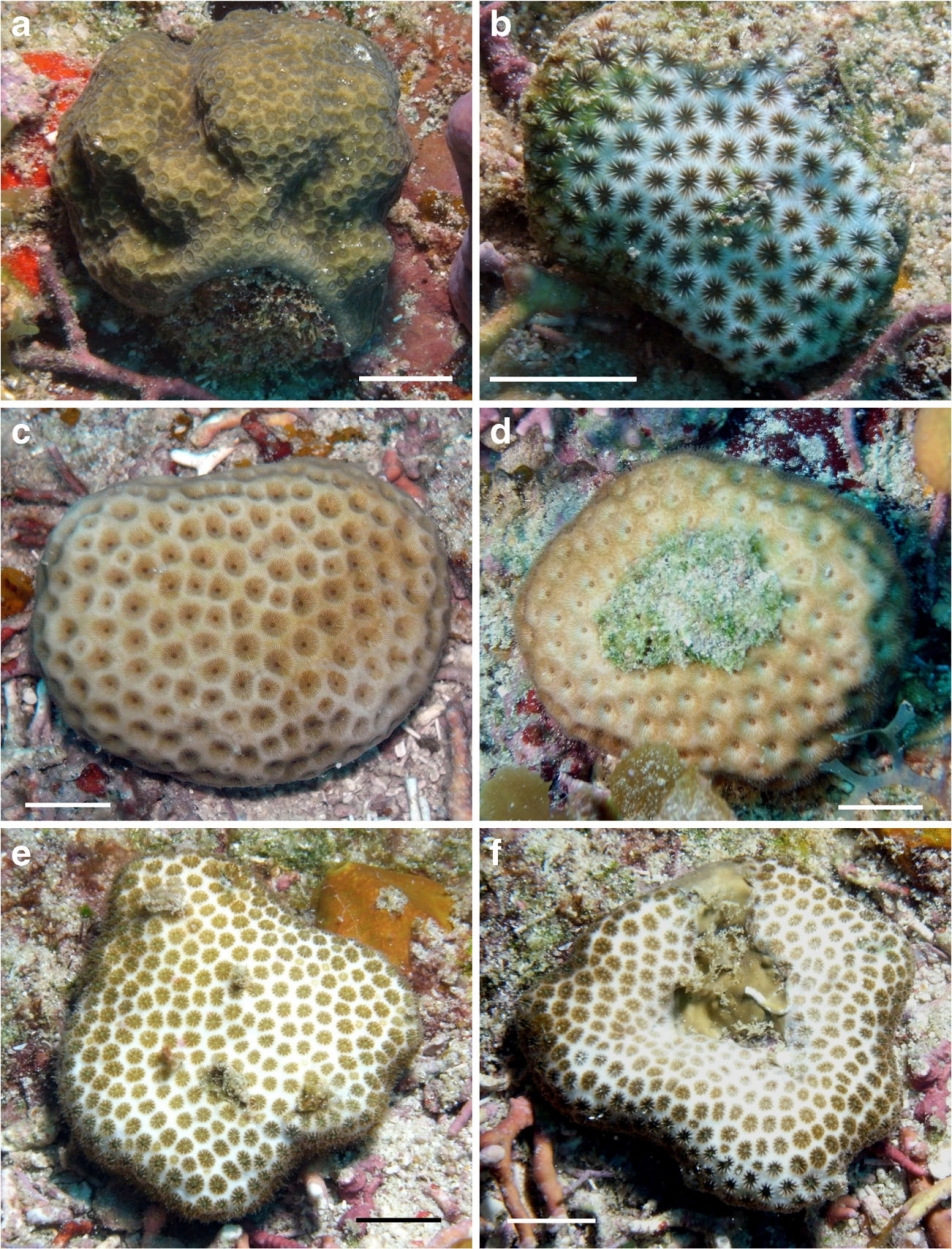
Spheroidal–amoeboidal coralliths (globular or massive shape). **a** *Porites astreoides.* **b** *Siderastrea radians*. **c**, **d** *S. siderea* (upper and lower sides). **e–f** *Stephanocoenia intersepta* (upper and lower sides). Locality: Tertre de Fleur, Saba Bank. *Scale bars* 1 cm

Corals of four species showed a predominantly spherical or amoeboidal coral shape (Table 2, Fig. 5): *Stephanocoenia intersepta* (*n = 2*), *Porites astreoides* (*n = 1*), *Siderastrea radians* (*n = 1*), and *S. siderea* (*n = 7*). Their maximum observed diameter was 5 cm (Fig. 5c, d). *Siderastrea radians* and *S. siderea* were previously known to form coralliths (Kissling 1973; Schuhmacher 1976; Lewis 1989; Sorauf and Harries 2009) as well as their congeners *S. stellata* Verrill, 1868 in Brazil (Lima and Coutinho 2016), *S. savignyana* Milne Edwards and Haime, 1850 in Madagascar, identified as *S. radians* by Pichon (1974), and *S. glynni* Budd and Guzmán, 1994 in the eastern Pacific (Budd and Guzman 1994), which turned out to be a *S. siderea* population introduced from the Atlantic (Glynn et al. 2016). Specimens of the massive scleractinian *P. astreoides* in Yucután, Mexico, were observed as epibionts on the axis of gorgonians, which eventually broke off, and in this way also became free living (Rodríguez-Martínez and Jordán-Dahlgren 1999). Other massive Caribbean species known to form coralliths but not found in free-living form during the present study are *Solenastrea bournoni* and *D. stokesii* (Kissling 1973). Both species have been recorded from Saba Bank (Table 1). *F. fragum* has also been mentioned as occurring in corallith shape, but no original source and locality were mentioned (Glynn 1974).

**Fig. 6:**
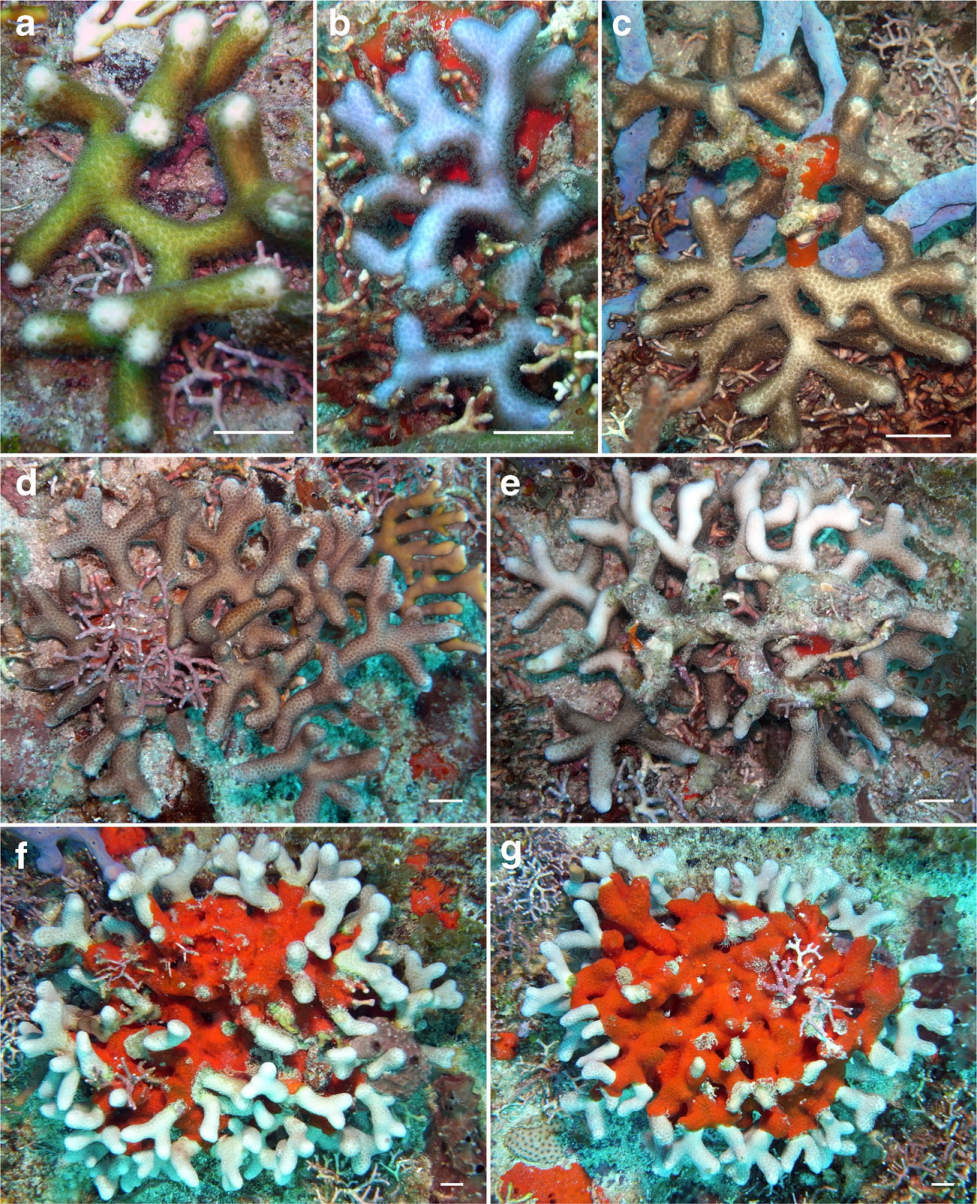
Various sizes of spheroidal ramose coralliths (tumbleweed shape) of *Porites divaricata*: small (**a**), medium (**c**), large: upper and lower sides shown (**d**, **e**); *P. furcata*: small (**b**), large: upper and lower sides shown (**f**, **g**). Locality: Tertre de Fleur, Saba Bank. *Scale bars* 1 cm

Free-living specimens of two species resembled tumbleweeds by showing long, slender branches directing in various directions, *Porites divaricata* and *P. furcata* (Table 2, Fig. 6). Fourteen of such specimens were found, and their maximum observed diameter was 21 cm (Fig. 6f, g). These species have previously not been described as coralliths. Their congener, *Porites sverdrupi* (Durham, 1947), an endemic of the Gulf of California, has been reported to show a similar kind of ramose corallith but its branches appear to be shorter, less straight, and more compactly arranged (Reyes-Bonilla et al. 1997; López-Pérez 2013; Paz-Garcia and Balart 2016). Examples of tumbleweed coralliths from the Indo-Pacific are, for example, regenerated branch fragments of *Acropora* (Riegl et al. 1996; Yusuf and Budiyanto 2012), *Montipora* (Shaish et al. 2010), *Pavona* (Scoffin et al. 1985), *Pocillopora* (Glynn 1974; Roff 2008), and *Psammocora* (Feingold 1996; Denis et al. 2015; Randall 2015).

**Fig. 7:**
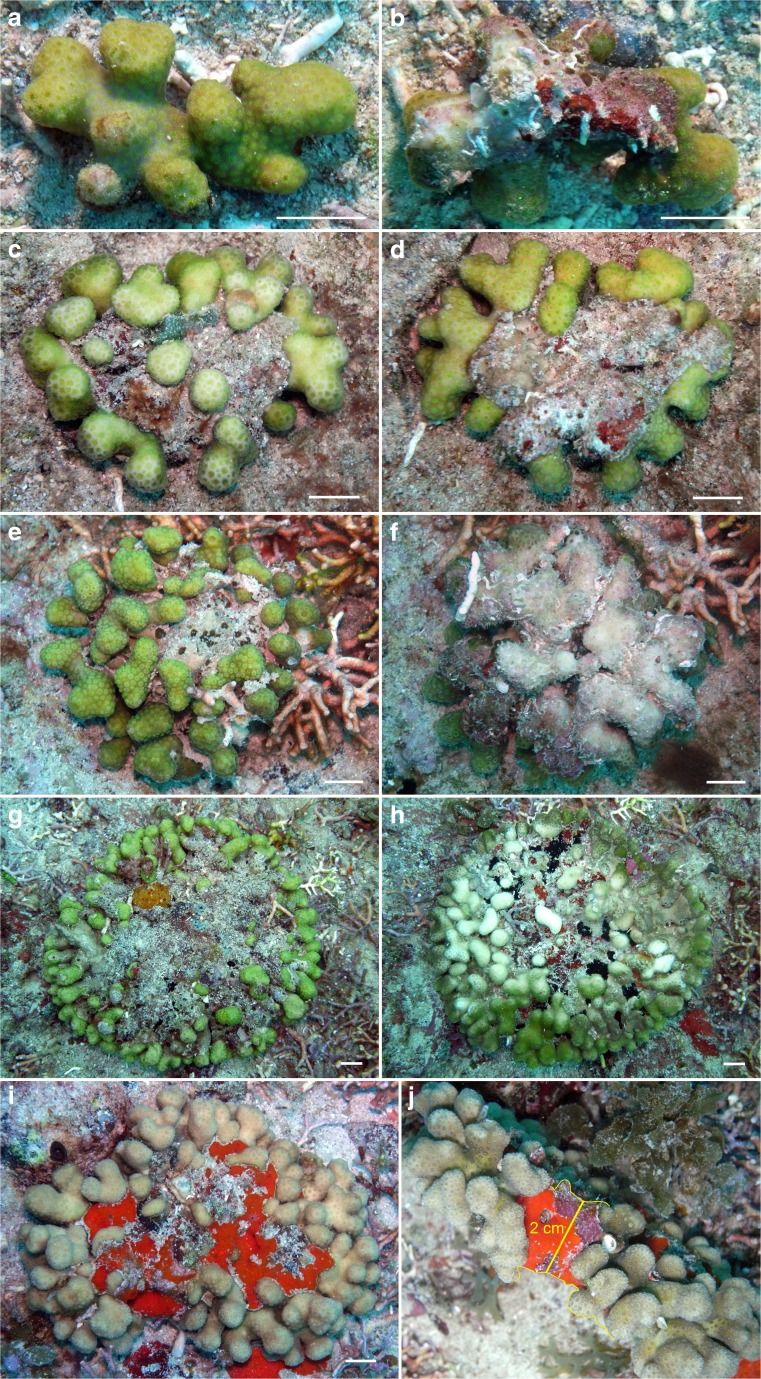
Various sizes of discoidal branched coralliths of *Madracis decactis*, upper and lower sides: small (**a**, **b**), medium (**c–f**), and large (**g, h**). Large specimens of *Madracis* cf. *auretenra*, upper side (**i**) and lateral side (**j**). Locality: Tertre de Fleur, Saba Bank. *Scale bars* 1 cm

Twelve out of 18 observed *Madracis* colonies appeared to be free living. Most of them belonged to *Madracis decactis*. Such coralliths showed a discoidal form when full grown, with short stubby branches. Their lower and upper surfaces showed less growth than their lateral periphery (Fig. 7). The lower surface was in direct contact with the substrate while the upper surface seemed to suffer from sediment smothering. Smaller coralliths consisted of loose fragments that were not flattened (Fig. 7a, b). The largest specimen was 14 cm wide (Fig. 7g, h). A 11.5-cm wide specimen was 2 cm thick at its centre (Fig. 7i, j). It may have belonged to *Madracis auretenra* because it was more ochre and not as green as regular *M. decactis* corals, and although *M. auretenra* (previously known as *Madracis mirabilis*) usually has thin, long branches that easily break (Bak and Criens 1981), it may also form thick and short branches (Fenner 1993; Bruno and Edmunds 1997). Both species have corallites with 10 septa and can normally be distinguished by coloration and branch shape (Bright and Lang 2013; Humann and DeLoach 2013). The latter species is commonly known as “yellow pencil coral” (Humann and DeLoach 2013), but it is unknown whether a yellow or ochre coloration is also possible in *M. decactis*. The flattened upper surface of the *Madracis* coralliths may be related to sediment accumulation between the short branches on top of the corals. Sediment was reported to negatively affect growth and survival in fragments of branching *Madracis* at Curaçao (Nagelkerken et al. 2000). In southeastern Brazil, *M. decactis* has been observed to form subspheroid coralliths up to 15 cm wide (Capel et al. 2012). The shape of discoidal coralliths resembles the form of large free-living mushroom corals (see Hoeksema and Matthews 2011; Hoeksema and Benzoni 2013) and some extinct scleractinian and non-scleractinian corals with analogue shapes (Gill and Coates 1977; Höfling 1989; Webb 1994; Scrutton 1996; Plusquellec et al. 1999; Pandey et al. 2011).

The Saba Bank assemblage of unattached corals consists of at least nine species (Table 2). Two of these are known to be represented by an anthocyathus free-living phase. In the other seven species, a corallith shape appears to happen by way of ecophenotypic variation, which may be most common in *Siderastrea* spp. Massive coralliths develop by forceful detachment from the sea floor or by overgrowing and incorporating a loose piece of substrate. In branching corals, corallith-forming can be a result of fragmentation and regeneration, which serves as a survival and reproduction strategy (Bak and Criens 1981; Highsmith 1982; Wallace 1985). It can also operate as dispersal mechanism if coral fragments do not self-attach (Guest et al. 2011) or fuse (Heyward and Collins 1985; Nothdurft and Webb 2012). Although a larger proportion of coral cover may be detached from the substrate than appears at first sight, its mobility may depend on how much consolidated the fragments are (Hoeksema 1988).

The occurrence of a multi-species assemblage of coralliths hints at a shared environmental connection. Assemblages of free-living coral species are known as colonizers of sandy substrates (Goreau and Yonge 1968; Fisk 1983; Hoeksema 2012b; Meesters et al. 2013). On the swell-exposed Saba Bank site, all coralliths were encountered on a more or less horizontal carbonate outcrop <20 m deep, where coral growth was limited in size and cover (Fig. 2). Wave force is known to break and dislodge sedentary organisms and also to limit their size (Dollar 1982; Denny et al. 1985; Madin et al. 2014). In various areas, wave action and currents have been recognized as the cause of corallith formation (Scoffin et al. 1985; Roff 2008; Sorauf and Harries 2009; Kersting et al. 2017a, 2017b), although in others places, bioturbation was seen as the driving force (Glynn 1974; Capel et al. 2012). Considering the swell and the apparent absence of burrowing animals, wave action imposed on Saba Bank has most likely caused some corals to break loose and to continue life as coralliths.

The presence of abundant unattached branching red coralline algae, forming rhodoliths and maerl deposits at Saba Bank, is consistent with this observation. Rhodoliths were not reported from similar depths (>15 m) at the more sheltered reefs of the adjacent island St. Eustatius (Van der Loos and Prud’homme van Reine 2016; Van der Loos et al. 2017). Actually a single rhodolith was found here (Van der Loos, pers. comm), which is a rare observation and therefore still consistent with the difference in wave exposure between Saba Bank and St. Eustatius.

Coral colonies are composed of multiple polyps that may continue to multiply by budding until the corallum has reached a maximum size. All observed coralliths show such a modular corallum architecture. Continuous fragmentation and regeneration may enhance their chances of survival (Highsmith 1982) and help them to postpone and perhaps overcome determinate growth caused by size-related physiological constraints (Hoeksema 1991). As unattached and mobile corals, they may undergo sessile dispersal over the sea floor and spread the risk of mortality (Jackson 1986). However, modular growth is not a condition for fragmentation because corals consisting of a single polyp can also break into fragments, regenerate, and continue life as unattached corals (Colley et al. 2002; Hoeksema and Waheed 2011b; Tokuda et al. 2017).

The present report indicates that Saba Bank offers a habitat to a rarely encountered assemblage of corals, which supports the idea that Saba Bank can serve an essential ecological role in the Eastern Caribbean and that it requires further conservation efforts (Hoetjes and Carpenter 2010; De Bakker et al. 2016).
